# IMPACT OF VALUE-BASED PURCHASE MODEL ON RACIAL DISPARITIES IN ACCESSING HIGH-QUALITY HOME HEALTH CARE

**DOI:** 10.1093/geroni/igae098.2060

**Published:** 2024-12-31

**Authors:** Chenjuan Ma, Shaohui Wu, Jason Fletcher

**Affiliations:** New York University, New York, New York, United States; Dartmouth College, Hanover, New Hampshire, United States; New York University, New York, New York, United States

## Abstract

Home healthcare (HHC) plays a pivotal role in serving Americans who “Age in Place”, particularly persons living with dementia (PLWD). Along with its rapid growth, there are increasing concerns of care disparities under the current HHC system and policies. This study aimed to examine racial/ethnic disparities in accessing high quality HHC among PLWD and identify the impact of home health value-based purchasing model (HHVBP), a policy designed for improving overall care quality, on such disparities. This study used two years of CMS HHC assessment data (i.e., OASIS) and HHC Compare Data. High quality HHC was identified based on agency care quality star rating (>=4.5 stars). HHVBP status was indicated by a dummy variable. A total of 743,860 PLWD from 6,410 agencies were studied, including 80.9% White, 11.9% Black, 5.6% Hispanic, and 1.6% Asian. Nearly one-third (32.6%) of agencies were with high quality care. Our risk-adjusted model shows that Blacks were 2% more likely to access high quality care while Hispanics and Asians were both about 20% less likely to access high quality care, compared to Whites. HHVBP increased the odds of accessing high quality HHC for all racial/ethnic groups (significant interaction terms all ORs>1) with most substantial among Hispanics (OR=1.60), followed by Asians (OR=1.28) and Blacks (OR=1.11). Stratification analysis indicated while the racial/ethnic disparities continue to exist in non-HHVBP states, such disparities disappeared in HHVBP states. Our study demonstrated the existence of racial/ethnic disparities in accessing high quality HHC and the effect of HHVBP in reducing such disparities.

